# Improvement of antibiotic activity of *Xenorhabdus bovienii* by medium optimization using response surface methodology

**DOI:** 10.1186/1475-2859-10-98

**Published:** 2011-11-14

**Authors:** Yonghong Wang, Xiangling Fang, Fengqiu An, Guohong Wang, Xing Zhang

**Affiliations:** 1 Research and Development Center of Biorational Pesticides, Northwest A & F University, Xinong Road 22, Yangling, Shaanxi 712100, P. R. China

## Abstract

**Background:**

The production of secondary metabolites with antibiotic properties is a common characteristic to entomopathogenic bacteria *Xenorhabdus* spp. These metabolites not only have diverse chemical structures but also have a wide range of bioactivities with medicinal and agricultural interests such as antibiotic, antimycotic and insecticidal, nematicidal and antiulcer, antineoplastic and antiviral. It has been known that cultivation parameters are critical to the secondary metabolites produced by microorganisms. Even small changes in the culture medium may not only impact the quantity of certain compounds but also the general metabolic profile of microorganisms. Manipulating nutritional or environmental factors can promote the biosynthesis of secondary metabolites and thus facilitate the discovery of new natural products. This work was conducted to evaluate the influence of nutrition on the antibiotic production of *X. bovienii* YL002 and to optimize the medium to maximize its antibiotic production.

**Results:**

Nutrition has high influence on the antibiotic production of *X. bovienii* YL002. Glycerol and soytone were identified as the best carbon and nitrogen sources that significantly affected the antibiotic production using one-factor-at-a-time approach. Response surface methodology (RSM) was applied to optimize the medium constituents (glycerol, soytone and minerals) for the antibiotic production of *X. bovienii* YL002. Higher antibiotic activity (337.5 U/mL) was obtained after optimization. The optimal levels of medium components were (g/L): glycerol 6.90, soytone 25.17, MgSO_4_·7H_2_O 1.57, (NH_4_)_2_SO_4_ 2.55, KH_2_PO_4_ 0.87, K_2_HPO_4_ 1.11 and Na_2_SO_4_ 1.81. An overall of 37.8% increase in the antibiotic activity of *X. bovienii* YL002 was obtained compared with that of the original medium.

**Conclusions:**

To the best of our knowledge, there are no reports on antibiotic production of *X. boviebii* by medium optimization using RSM. The results strongly support the use of RSM for medium optimization. The optimized medium not only resulted in a 37.8% increase of antibiotic activity, but also reduced the numbers of experiments. The chosen method of medium optimization was efficient, simple and less time consuming. This work will be useful for the development of *X. bovienii* cultivation process for efficient antibiotic production on a large scale, and for the development of more advanced control strategies on plant diseases.

## Background

*Xenorhabdus* is a unique genus of bacteria, the species (strains) of which are symbiotically associated with entomopathogenic nematode belonging to the genus *Steinernema*. The primary (1°) forms of the bacteria are carried in the intestine of the infective dauer juvenile (IJ) developmental stage of the nematode. The IJ penetrates an insect host and releases the bacteria into the hemocoel of the host. The bacteria multiply rapidly and produce various metabolites which can overcome the insect immune system [1], kill the insect, and inhibit the growth of various fungal and bacterial competitors [2-4]. By doing so, the bacterial symbionts are believed to prevent putrefaction of the insect cadaver and establish conditions that favor the development of both the nematode and bacterial symbionts [5]. The antimicrobial nature of metabolites produced by *Xenorhabdus* spp. is known, and several compounds with antibiotic activity have been isolated and identified. These include indoles [6], xenorhabdins [7], xenocoumacin [8], nematophin [9], benzylineacetone [10], xenortides and xenematide [11], and cyclolipopeptide [12]. These metabolites not only have diverse chemical structures, but also have a wide range of bioactivities with medicinal and agricultural interests, such as antibiotic, antimycotic, insecticidal, nematicidal, antiulcer, antineoplastic and antiviral.

*X. bovienii* has been known to produce two classes of antibiotics, indoles and dithiolopyrrolones (xenorhabdins, xenomins and xenorxides), which could inhibit the growth of *Botrytis cinerea*, *Phytophthora capsici*, and *P. ultimum*[13]. *X. bovienii* strain A2 appears to be unique in the diversity of small-molecule antimicrobial compounds since four indoles, several xenorhabdins, xenomins and xenorxides have been isolated from this strain alone [14]. These compounds showed strong activity against Gram-positive bacteria, yeast and many fungal species. It was concluded on the basis of *in vitro* tests that the antibiotics from *X. bovienii* may offer a good opportunity for the control of diseases caused by some species of plant pathogenic fungi. *X. bovienii* metabolites can suppress *P. infestans* on potato leaves with only slight phytotoxicity [15]. The metabolites from *X. bovienii* exhibited 100% inhibition effect on *P. cactorum* lesions of pecan leaves [16]. Similarly, we also found that the methanol extracted bioactive compounds from *X. bovienii* YL002 showed potent therapeutic and protective effects against *B. cinerea* on tomato plants and *P. capsici* on pepper plants [17].

It has been known that cultivation parameters are critical to the secondary metabolites produced by microorganisms. Even small changes in the culture medium may not only impact the quantity of certain compounds but also the general metabolic profile of microorganisms [18]. In particular, in the field of antibiotics, much effort was directed toward optimizing production rates and directing the product spectrum. Manipulating nutritional or environmental factors can promote the biosynthesis of secondary metabolites and thus facilitate the discovery of new natural products. Antibiotic production by *Xenorhabdus* spp. differs qualitatively and quantitatively depending on the strains and species of bacteria and their culture conditions. No antibiotic activity was detected, using an agar diffusion assay, from the metabolites of *Xenorhabdus* spp. cultured in 1% peptone water. However, other media have been used successfully for antibiotic production, including yeast extract broth and its modifications [2,19], Luria-Bertani broth [19], sea water medium [6] and TSB [9,10]. These results indicate that nutrition plays an important role in the onset and intensity of secondary metabolites, not only because limiting the supply of an essential nutrient is an effective means of restricting growth but also because the choice of limiting nutrient can have specific metabolic and regulatory effects [20].

Recently, whole-genome sequencing programmes have revealed that the biosynthetic potential of microorganisms has been greatly underexplored, relying as it does on traditional approaches. In fact, the number of genes encoding biosynthetic enzymes in various bacteria including *X. nematophila* ATCC19061, the best studied strain of these bacterial symbionts, clearly outnumbers the known secondary metabolites of these organisms [21]. The majority of these encoded molecules are cryptic. One reason for this observation might be that only a subset of biosynthetic pathway genes is expressed under standard laboratory culture conditions and therefore only a minority of potential chemical structures is produced. Most cryptic metabolite biosyntheses are tightly regulated, and are only activated under specific conditions. The methods to trigger biosynthetic pathways to yield cryptic natural products involve the culture conditions, external cues, co-cultivation and genomic approaches such as genome-mining, epigenetic remodeling, and engineered pathway activation [18]. For example, altering easily accessible cultivation parameters, such as media composition, aeration, temperature or shape of culturing flask, can isolate hitherto unknown natural products from various fungi and actinomycetes [22]. Therefore, to promote the biosynthesis of secondary metabolites and facilitate the discovery of new natural products, it is a prerequisite to design a proper production medium in an efficient fermentation process.

Response surface methodology (RSM) is a commonly used method to find the optimal fermentation conditions for various microorganisms, and also an efficient statistical technique for optimization of multiple variables with minimum number of experiments [23-26]. The main advantage of RSM is the reduced number of experimental runs needed to provide sufficient information for statistically acceptable result. It is a faster and less cost method for gathering research result than the classical methods. The main aim of this work was to optimize the medium to maximize antibiotic production by *X. bovienii* YL002, a strain isolated from its nematode symbiont *Steinernema* sp YL002, which was obtained from the soil of Yangling, China. A stepwise optimization strategy was performed (1) screening the original medium; (2) selecting the best carbon and nitrogen sources that significantly affect antibiotic production using one-factor-at-a-time approach; (3) RSM optimization of these significant ingredients by central composite design (CCD).

## Results and Discussion

### Effect of different media on growth and antibiotic activity

**Figure 1:**
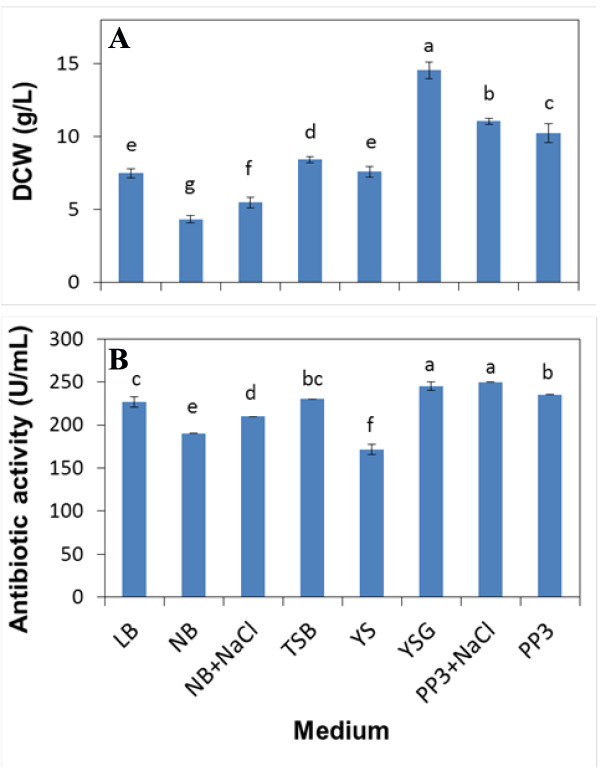
**The effect of different media on DCW (A) and antibiotic activity (B) of *Xenorhabdus bovienii* YL002**.

The different medium had significant effects on cell growth and antibiotic activity of *X. bovienii* YL002 (Figure 1). The modified PP3 broth (PP3+NaCl) showed a maximum antibiotic activity (250.0 U/mL), followed by YSG medium (245.0 U/mL), PP3 broth (235.0 U/mL), TSB medium (230.0 U/mL), LB medium (226.7 U/mL), modified nutrient broth (NB+NaCl) (210.0 U/mL), nutrient broth (NB) (190.0 U/mL) and YS medium (171.7 U/mL). The modified PP3 broth and YSG medium showed the similar antibiotic activity. In addition, YSG medium also showed the maximum DCW as compared with the others.

YSG medium was the superior medium for higher antibiotic activity and biomass of *X. bovienii* YL002, which is in good agreement with previously reports which assert that YSG medium was able to support relatively high antibiotic production of *Xenorhabdus* spp. [19,27]. Hence, YSG medium was employed as an original medium for the carbon and nitrogen sources selection studies.

Osmolarity is an underlying physiological parameter which is associated with the virulence of bacteria [28]. When osmolarity in the medium was increased by the addition of NaCl, *X. bovienii* YL002 drastically increased metabolite production, the antibiotic activity and DCW in PP3+NaCl and NB+NaCl are significant higher than that in PP3 and NB (Figure 1). Similar results were obtained when altering the osmolarity using NaCl, the metabolic profiles were significantly altered in *X. nematophila*[29]. Increased osmotic stress by salt generally stimulated metabolite production in *X. nematophila* cultures [29]. When osmolarity was altered by the addition of the solute sorbitol, *X. nematophila* cultures exhibited reduced metabolite production and poor growth characteristics [29]. This reciprocal production profile suggests that osmolarity does contribute to the metabolic shift, which support the notion that hypoosmotic conditions were previously described to maintain a stable secondary form (2°) that does not produce antibiotics in rich medium [30].

### Effect of various carbon and nitrogen sources on antibiotic activity

**Figure 2:**
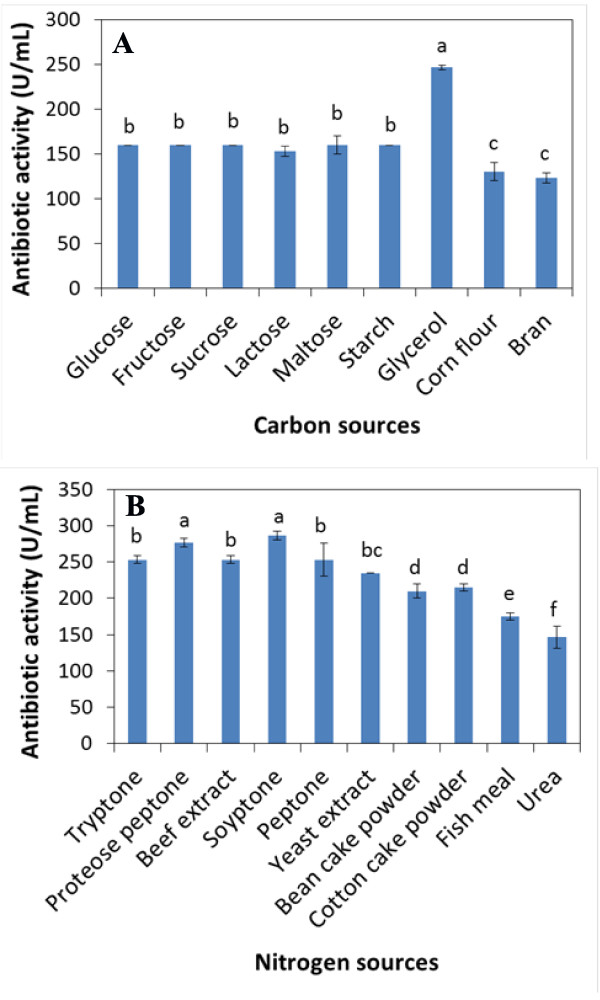
**The effect of different carbon (A) and nitrogen (B) sources on antibiotic activity of *Xenorhabdus bovienii* YL002**.

The carbon and nitrogen sources are the important constituents to be considered which are reported to have highly influence on the antibiotic production by *Xenorhabdus* spp. [27,31,32]. Based on YSG medium, the effect of various carbon and nitrogen sources on antibiotic activity of *X. bovienii* YL002 were studied by employing these at a final concentration of 5 g/L and 15 g/L for carbon and nitrogen sources, respectively. Results are presented in Figure 2. The results indicated that among the various carbon sources studied, the *X. bovienii* YL002 produced the maximum antibiotic activity in glycerol (246.7 U/mL). The antibiotic activity in glucose, fructose, maltose, sucrose, lactose and starch ranged from 153.3 to 160.0 U/mL. The antibiotic activity with the addition of crude carbon sources (corn flour) was 130.0 U/mL, and the minimum (123.3 U/mL) in bran. As for the various nitrogen sources, soytone (286.7 U/mL) and proteose peptone (276.7 U/mL) favored antibiotics production, and the antibiotic activity had no significant differences. Lower antibiotic activities were observed with urea and crude nitrogen sources (bean cake powder, cotton cake powder and fish meal). Thus, glycerol and soytone were chosen as the source of carbon and nitrogen for further experiments, respectively. Similar results were obtained in the study on the determination of the effect of various carbon sources and nitrogen sources on the antibiotic activity of *Xenorhabdus* sp. D43, maltose and glycerol had the strongest effect on antibiotic activity of the strain [31]. However, previous study found that the optimal carbon sources and nitrogen sources for antibiotics production by *X. nematophila* BJ was glucose and soytone [32]. For YL001 and TB strain of *X. nematophila*, the optimal carbon sources and nitrogen sources for antibiotics production was glucose and peptone [27,33]. The disparity may be due to the use of different bacterial strains and different culture condition. It is also conceivable that the difference may be due to different original medium for carbon and nitrogen sources selection.

It is known that several trace elements are essential for microbial growth because of their involvement in metalloenzymes or as enzyme activators. In secondary metabolism, zinc, iron and manganese are the most important trace elements [34]. MgSO_4_, MgCl_2_, NaCl, KH_2_OP_4_, KNO_3_ and (NH_4_)_2_SO_4_ favored antibiotic production by *Xenorhabdus* sp. D43, but antibiotic production with the addition of Zn(NO_3_)_2_ and CuSO_4_ was found to be low [31]. In YSG, the minerals contain MgSO_4_, (NH_4_)_2_SO_4_, KH_2_OP_4_, K_2_HOP_4_ and Na_2_SO_4_. Therefore, the minerals for further CCD design were defined as the total concentration of the minerals of YSG medium and kept the proportion of diversified components. Subsequently, experiments were conducted for further optimization of these selected nutrients employing RSM.

### Optimization of medium constituents

#### ANOVA and model fitting

After determination of carbon and nitrogen sources in the medium, the effects of concentration of medium constituents on the antibiotic activity with the producer YL002 were further investigated. The range and the levels of the variables investigated in this study are given in Table 1. A total of 20 experiments with different combination of glycerol, soytone, and minerals were performed, and the results of experimental for studying the effects of three independent variables, viz., glycerol, soytone, and minerals, on antibiotic activity are presented along with the observed and predicted response, which shows considerable variation in the antibiotic activity depending on the three independent variables in the medium (Table 2). Treatment run 1, 5, 9, 11 and 15-20 showed a high antibiotic activity (≥ 280 U/mL). The maximum antibiotic activity (305.0 U/mL) was achieved in run number 1, while the minimum antibiotic activity (230.0 U/mL) was observed in run number 14. Treatment runs 15-20 were the center points in the design, which were repeated six times for estimation of error. This result suggests that the data was deviated and the flask experiments were accurate.

**Table 1:** Experimental range and level of the independent variables

Variable	Parameter	Range and level				
	
		-1.682	-1	0	1	1.682
*X*_1_	Glycerol (g/L)	1.0	3.0	5.0	7.0	9.0
*X*_2_	Soytone (g/L)	5.0	9.0	15.0	21.0	25.0
*X*_3_	Minerals (g/L)	3.0	4.2	6.2	8.2	9.4

*x_i_* = coded value of the variable *X_i_*.

*x*_1_ = (Glucose - 5)/2; *x*_2_ = (Peptone - 15)/6; *x*_3_ = (Minerals - 6.2)/2.

**Table 2:** CCD plan of coded value, the observed value and predicted value

Run	*X*_1_	*X*_2_	*X*_3_	Observed value (U/mL)	Predicted value (U/mL)	Residual
1	1	1	1	305.0	300.59440	4.40560
2	1	1	-1	270.0	269.50019	0.49981
3	1	-1	1	266.7	263.28293	3.41707
4	1	-1	-1	271.7	264.68872	7.01128
5	-1	1	1	280.0	278.74159	1.25841
6	-1	1	-1	250.0	245.14737	4.85263
7	-1	-1	1	241.7	233.93012	7.76988
8	-1	-1	-1	236.7	232.83590	3.86410
9	1.682	0	0	285.0	290.12933	-5.12933
10	-1.682	0	0	238.4	244.96290	-6.56290
11	0	1.682	0	290.0	292.56256	-2.56256
12	0	-1.682	0	241.7	250.82967	-9.12967
13	0	0	1.682	256.7	262.73135	-6.03135
14	0	0	-1.682	230.0	235.66088	-5.66088
15	0	0	0	280.0	291.33368	-11.33368
16	0	0	0	296.7	291.33368	5.36632
17	0	0	0	300.0	291.33368	8.66632
18	0	0	0	283.3	291.33368	-8.03368
19	0	0	0	290.0	291.33368	-1.33368
20	0	0	0	300.0	291.33368	8.66632

In order to evaluate the relationship between dependent and independent variables and to determine the maximum antibiotic activity production corresponding to the optimum levels of glycerol (*X*_1_), soytone (*X*_2_) and minerals (*X*_3_), a second-order polynomial model (Eq.2) was proposed to calculate the optimum levels of these variables. By applying the multiple regression analysis on experimental data, a second-order polynomial model in coded unit explains the role of each variable and their second-order interactions in antibiotic activity.

Estimated regression coefficients of the second-order polynomial model are shown in Table 3. The quadratic model of response equation in terms of coded factors is:

**Table 3:** Parameter estimates for factorial design experiments.

Effect	Parameter estimate	Standard error	Computed *t*-value	*P*-value
Intercept	291.3337	3.607650	80.75441	0.000000
*X*_1_	13.4264	2.393452	5.60964	0.000225
*X*_1_^2^	-8.4081	2.329651	-3.60917	0.004775
*X*_2_	12.4057	2.393452	5.18320	0.000411
*X*_2_^2^	-6.9412	2.329651	-2.97951	0.013819
*X*_3_	8.0471	2.393452	3.36213	0.007216
*X*_3_^2^	-14.8942	2.329651	-6.39332	0.000079
*X*_1_*X_2_	-1.8750	3.127355	-0.59955	0.562145
*X*_1_*X_3_	-0.6250	3.127355	-0.19985	0.845604
*X*_2_*X_3_	8.1250	3.127355	2.59804	0.026581

(3) $$\begin{array}{c} Y=291.3337+13.4264x_{1}+12.4057x_{2}+8.0471x_{3}\\ -1.875x_{1}x_{2}-0.625x_{1}x_{3}+8.125x_{2}x_{3}\\ -8.4081{x_{1}}^{2}-6.9412{x_{2}}^{2}-14.8942{x_{3}}^{2} \end{array}$$

where *Y* is the response, that is, the antibiotic activity units and *x*_1_, *x*_2_ and *x*_3_ are the coded values of the independent variables, viz., glucose, peptone and minerals, respectively. The quadratic model in Eq. (3) with 10 terms contains three linear terms, three quadratic terms and three two factorial interactions. After the neglect of insignificant terms (on the basis of *P*-values which were more than 0.05), the model Eq. (3) was modified to reduced fitted model Eq. (4):

(4) $$\begin{array}{c} Y=291.3337+13.4264x_{1}+12.4057x_{2}+8.0471x_{3}\\ +8.125x_{2}x_{3}-8.4081{x_{1}}^{2}-6.9412{x_{2}}^{2}-14.8942{x_{3}}^{2} \end{array}$$

**Figure 3:**
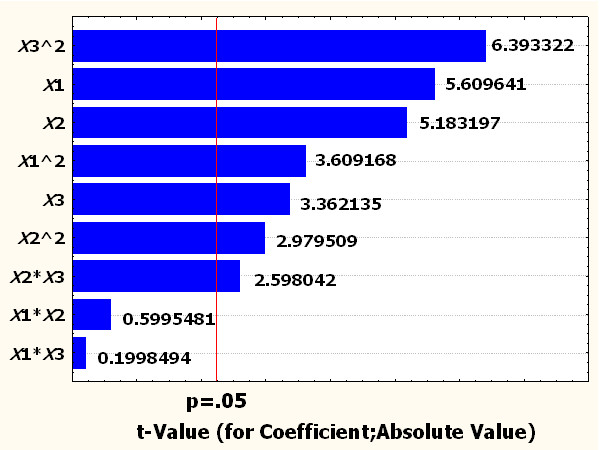
**Pareto chart of *t*-values for coefficients**.

Quadratic response surface regression designs are a hybrid type of design with characteristics of both polynomial regression designs and fractional factorial regression designs. Quadratic response surface regression designs contain all the same effects of polynomial regression designs to degree 2 and additionally the 2-way interaction effects of the predictor variables. Each effect could be estimate independently due to the orthogonal design. In order to evaluate influential factors/terms in the quadratic RSM, an ANOVA was established, and it can be seen from the degree of significance that the linear term of *X*_1_, *X*_2_ and *X*_3_ were significant at 5% level (Table 4). Among the linear terms, the main effects of glycerol and soytone on the antibiotics activity were highly significant as was evident from their respective *P*-values (*P_X_*_1_ = 0.000224 and *P_X_*_2_ = 0.000411). Minerals also had significant effect on the antibiotics activity at linear terms (*P_X_*_3_ < 0.007216). Partial eta squared was used as a measure of effect size. Glycerol had highest impact on the antibiotics production as given by highest linear partial eta squared (0.758851), followed by soytone (0.728744) and minerals (0.530603). The result also could be explained by the Pareto chart of effects (Figure 3). The Pareto chart shows each of the estimated effects, interactions and the standard error of each of the effects, which measures their sampling error. In the experimental design the Pareto chart is a Frequency Histogram that shows the amount of influence each factor has on the response in decreasing order, and often, a line going across the columns indicates how large an effect has to be (i.e., how long a column must be) to be statistically significant [35]. Quadratic term of *X*_1_^2^, *X*_2_^2^ and *X*_3_^2^ were significant at 5% level. The result also indicated that glycerol, soytone and minerals could act as limiting factors, and small variations in their values will considerably alter either growth rate or product formation rate or both. Interactive terms of *X*_2_*X*_3_ were also significant at 5% level. The interaction between soytone and minerals had significant effects on the antibiotics production (*P_X_*_2*X*3_ < 0.026581). These suggest that the concentrations of glycerol, soytone and minerals have a direct relationship with the antibiotic activity in this particular complex medium. The result fitted in the previous work, in which glycerol, soytone and minerals influence bacterial growth and antibiotic production [27,31-33].

**Table 4:** Univariate tests of significance, effect sizes, and powers for antibiotic activity sigma-restricted parameterization and effective hypothesis decomposition

Effect	SS	DF	MS	*F*	*P*	Partial eta-squared	Non- centrality	Observed power (alpha = 0.05)
Intercept	510243.0	1	510243.0	6521.276	0.000000	0.998469	6521.276	1.000000
*X*_1_	2462.2	1	2462.2	31.468	0.000225	0.758851	31.468	0.998878
*X*_1_^2^	1019.2	1	1019.2	13.026	0.004775	0.565710	13.026	0.900843
*X*_2_	2102.0	1	2102.0	26.866	0.000411	0.728744	26.866	0.996323
*X*_2_^2^	694.6	1	694.6	8.877	0.013819	0.470268	8.877	0.765827
*X*_3_	884.5	1	884.5	11.304	0.007216	0.530603	11.304	0.856889
*X*_3_^2^	3198.1	1	3198.1	40.875	0.000079	0.803438	40.875	0.999909
*X*_1_*X_2_	28.1	1	28.1	0.359	0.562145	0.034699	0.359	0.084539
*X*_1_*X_3_	3.1	1	3.1	0.040	0.845604	0.003978	0.040	0.053780
*X*_2_*X_3_	528.1	1	528.1	6.750	0.026581	0.402979	6.750	0.649570
Error	782.4	10	78.2					

The significance of each coefficient in this study was determined by Student's *t*-test and *P*-value. The larger the magnitude of *t*-test and smaller the *P*-value, the more significant is the corresponding coefficient. The positive coefficients for *X*_1_, *X*_2_ and *X*_3_ indicate a linear effect to increase antibiotic activity. The negative coefficient for *X*_1_^2^, *X*_2_^2^and *X*_3_^2^ showed negative effects on the antibiotics production, indicating that the antibiotics production increased as the level of these factors increased and decreased as the level of these parameters increased above certain values.

The results of the second-order response surface model in the form of analysis of variance (ANOVA) are given in Table 5. The ANOVA of the quadratic regression model demonstrated that the model was highly significant, as was evident from the low *P* value of the Fisher's *F*-test (*F*_model_, mean square regression/mean square residual = 21.52501, *P*_model_ >*F* = 0.000007]. Moreover, the computed *F*-value was much greater than the tabulated *F*-value (*F*_0.01 (7, 12)_ = 4.64), indicating that the treatment differences were highly significant. This proved that the model equation as expressed in Eq. (4) provides a suitable model to describe the response of the experiment pertaining to antibiotic activity. The model also showed a statistically insignificant lack of fit, as is evident from the lower calculated *F*-value (0.843414) than the tabulated *F*-value (*F*_0.01 (7, 5)_ = 10.5) even at the 0.01 confidence level. The model was found to be adequate for prediction within the range of variables employed. The goodness of fit of the model based on RSM can be checked by the coefficient of determination (*R*^2^), which provides a measure of how much variability in the observed response values can be explained by the experimental factors and their interactions. The *R*^2^ value is always between 0 and 1. The closer the *R*^2^ value is to 1.00, the stronger the model is and the better it predicts the response. In this case, the coefficient of determination *R*^2^ = 0.926233, which implied that antibiotic activity was attributed to the given independent variables. The *R*^2^ also indicated that only 7% of the total variations were not explained by the model. The value of the adjusted determination coefficient (adjusted *R*^2^ = 0.883203) was also high to indicate a high significance of the model. These measures indicated that the accuracy and general ability of the polynomial model was good and that analysis of the response trends using the model was reasonable. A higher value of the correlation coefficient, *R* = 0.96241, indicates a good agreement between the experimental and predicted values of antibiotic activity. These measures indicate that the accuracy and general ability of the polynomial model are good and that analysis of the response trends using the model is reasonable.

**Table 5:** Analysis of variance (ANOVA) of the quadratic model

Source of variations	DF	SS	MS	*F*-value	*P* >*F*
Model	7	10216.7500	1459.53600	21.525010	0.000007**
Residual	12	813.6782	67.80652		
Lack of fit	7	440.5649	62.93784	0.843414	0.596740
Pure error	5	373.1133	74.62267		
Total	19	11030.4282			

*R* = Coefficient of correlation = 0.962410; *R*^2^ = Coefficient of determination = 0.926233; adjusted *R*^2^ = 0.883203.

** Significant at 1% level.

**Figure 4:**
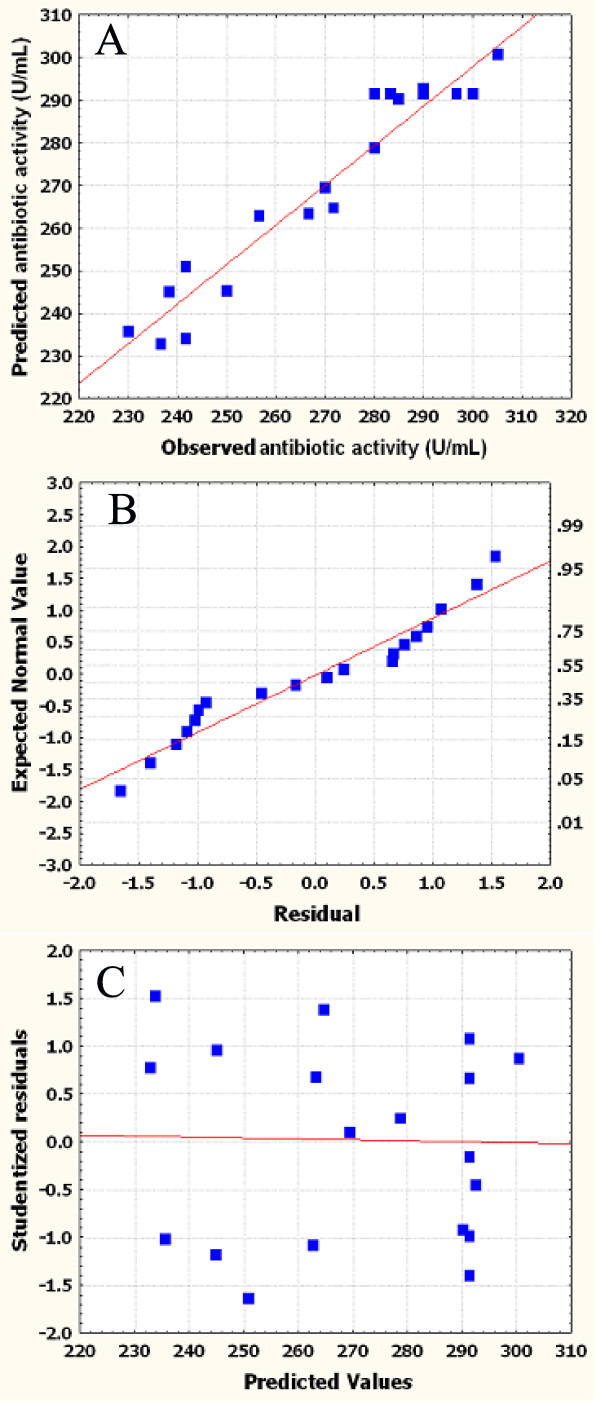
**Residual diagnostics of the contour surface of the quadratic model**. A) The predicted vs. observed by replicates of antibiotic activity of *Xenorhabdus bovienii* YL002. B) Normal probability of internally studentized residuals. C) Plot of internally studentized residuals vs. predicted response.

A regression model can be used to predict future observations on the response *Y* (antibiotic activity) corresponding to particular values of the regress variables. In predicting new observations and in estimating the mean response at a given point, one must be careful about extrapolating beyond the region containing the original observations. It is very possible that a model that fits well in the region of the original data will no longer fit well outside the region. Figure 4A shows observed antibiotic activity (the response) versus those from the empirical model Eq. (2). Point above or below the diagonal line represented areas of over or under prediction. The figure proves that no significant violations of the model were found in the analysis, and the predicted data of the response from the empirical model is in agreement with the observed ones in the range of the operating variables.

Usually, it is necessary to check the fitted model to ensure that it provides an adequate approximation to the real system. Unless the model shows an adequate fit, proceeding with the investigation and optimization of the fitted response surface likely give poor or misleading results. The residuals from the least squares fit play an important role in judging model adequacy. The assumptions for randomness, normality and constant variances of the residuals are all verified by the normal probability plot and the residual plot. The normal plot of residuals is shown in Figure 4B, as the most important diagnostic for the model, the normal probability plot of the residuals, came up by default. A linear pattern demonstrated normality in the error term, i.e., there were no signs of any problems in our data. Figure 4C presents a plot of residuals versus the predicted response. The general impression is that the residuals scatter randomly on the display, suggesting that the variance of the original observation is constant for all values of *Y*. Both of the plots are satisfactory, so we conclude that the empirical model is adequate to describe the antibiotic activity by response surface.

### Response surface analysis

The 3D response surface and the 2D contour plots described by the regression model are drawn to illustrate the effects of the independent variables, and interactive effects of each independent variable on the response variables. The shape of the corresponding contour plots indicates whether the mutual interactions between the independent variables are significant or not. An elliptical nature of the contour plots indicates that the interactions between the independent variables were significant. From the 3D response surface plots and the corresponding contour plots, the optimal values of the independent variables and the corresponding response could be predicted, and the interaction between each independent variable pair could be understood. The maximum predicted value is indicated by the surface confined in the smallest ellipse in the contour diagram.

**Figure 5:**
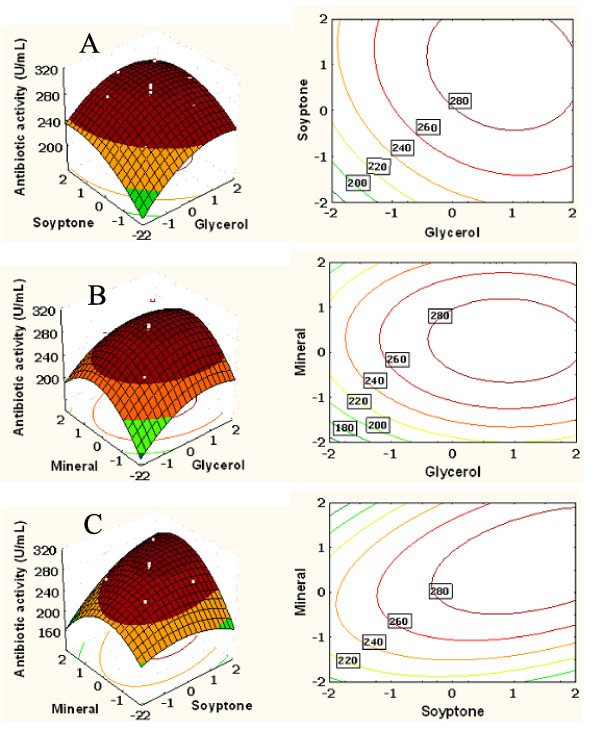
**Response surface plot and contour plot**. A) The combined effects of glycerol and soytone on the antibiotic activity of *Xenorhabdus bovienii* YL002. B) The combined effects of glycerol and minerals on the antibiotic activity of *X. bovienii* YL002. C) The combined effects of soytone and minerals on the antibiotic activity of *X. bovienii* YL002.

Figure 5A showed the 3D plot and its corresponding contour plot, showing the effects of glycerol and soytone on the antibiotic activity, while the minerals was fixed at its middle level. Glycerol has no significant interaction with soytone, which is evident from the relatively circular nature of the contour curves. With the increase of the concentration of soytone from 5.0 to 21.0 g/L (coded value -1.682 to 1.0), the antibiotic activity significantly increased from 199.16 to 253.58 U/mL at a low concentration of glycerol (-1.682), and increased from 254.91 to 292.44 U/mL at a high level of glycerol (1.682). When the concentration of glycerol was near 7.0 g/L (1.0), increasing the concentration of soytone from 5.0 to 21.0 g/L to some extent favored the accumulation of antibiotic, and any further increase in its values resulted in decreased antibiotic activity. Also, When the concentration of soytone was near 21.0 g/L (1.0), increasing the concentration of glycerol from 1.0 to 7.0 g/L (coded value -1.682 to 1.0) to some extent favored the accumulation of antibiotic, and any further increase in its values resulted in decreased antibiotic activity. The three-dimensional plot and its respective contour plot facilitated the identification of the optimum levels of glycerol and soytone. The analysis of Figure 5A reveals that the optimal concentration of glycerol was around 7.0 g/L, and the concentration of soytone was around 21.0 g/L.

Figure 5B depicts the 3D plot and its corresponding contour plot showing the effects of glycerol and minerals on the antibiotic activity, while the soytone were fixed at its middle level. There was insignificant mutual interaction between glycerol and minerals, which is also evident from the relatively circular nature of the contour curves. When the concentration of glycerol was near 7.0 g/L (1.0), increasing the concentration of minerals from 3.0 to 6.7 g/L (-1.682 to 0.25) to some extent favored the accumulation of antibiotic, the antibiotic activity increased from 240.68 to 279.43 U/mL, and any further increase in its values resulted in decreased antibiotic activity. With the increase of the concentration of minerals from 3.0 g/L to 6.7 g/L, the antibiotic activity significantly increased from 195.08 to 246.03 U/mL at a low concentration of glycerol (-1.682), and increased from 234.16 to 291.21 U/mL at a high level of glycerol (1.682). The analysis of Figure 5B reveals that the optimal concentration of minerals was around 6.7 g/L.

Figure 5C presents 3D plot and its corresponding contour plot showing the effects of soytone and minerals on the antibiotic activity, while the concentration of glycerol was fixed at its middle level. There was a significant mutual interaction between soytone and minerals. Under the moderate concentration of minerals, the antibiotic activity increased with increasing the concentration of soytone from 5.0 to 21.0 g/L (coded value -1.682 to 1.0), and any further increase in its values resulted in decreased antibiotic activity. At a high level of minerals (1.682), the antibiotic activity steadily increased when increasing the concentration of soytone. However, no significant effect of the concentration of soytone on antibiotic activity was observed at a lower level of minerals.

### Optimization of response

**Figure 6:**
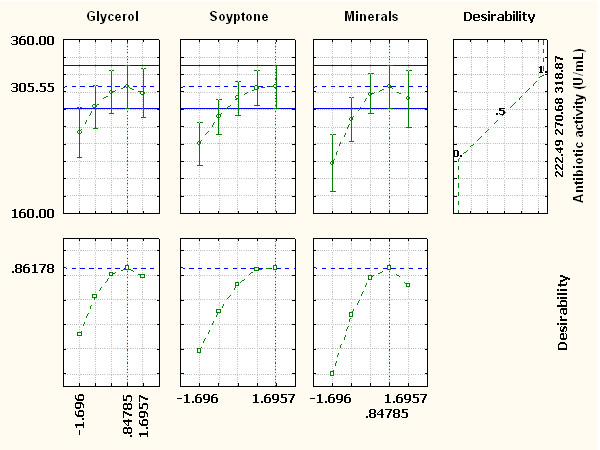
**Desirability charts of variables for maximum response**.

The desirability function to obtain an optimum antibiotic activity was fitted by the least square method assigning the antibiotic activity at the observed low (222.49 U/mL) and high (318.87 U/mL) values for a corresponding desirability of 0 and 1, respectively, and the profiles were plotted using Response Surface Regression in STATISTICA 8.0 software. These desirability profiles show which levels of predictor (*X*_1_, *X*_2_ and *X*_3_) variables produce the most desirable predicted responses on the dependent variable (*Y*). The profiles for predicted response and the desirability level for factors (Figure 6) indicate that glycerol 6.9 g/L, soytone 25.17 g/L and mineral 7.9 g/L (the minerals were translated into the different components of YSG minerals according to the proportion. The values (MgSO_4_·7H_2_O 1.57 g, (NH_4_)_2_SO_4_ 2.55 g, KH_2_PO_4_ 0.87 g, K_2_HPO_4_ 1.11 g, Na_2_SO_4_ 1.81 g) give optimum antibiotic activity at an optimum desirability score of 0.86178 (305.55 U/mL). These profiles suggest that an increase in the concentration of glycerol, soytone and mineral above the optimized levels will not increase the antibiotic activity significantly. The verification of the results using the optimized medium was accomplished by carrying out shake-flask experiments. The maximum antibiotic activity unit obtained experimentally was found to be 337.5 U/mL. This is obviously in close agreement with the model prediction (305.55 U/mL). As a result, the model developed was considered to be accurate and reliable for predicting the production of antibiotics by *X. bovienii* YL002. After optimization, the antibiotic activity was improved by 15.7% and 37.8%, compared with mean observed response and at zero level of all variables (291.7 U/mL) and YSG medium (245.0 U/mL).

## Conclusion

Statistical optimization method for the fermentation process can overcome the limitations of classic empirical methods and was proved to be a powerful tool for the optimization of antibiotics production by *X. bovienii* YL002. In the present study, glycerol and soytone were identified as the most important components for enhancing antibiotic production by *X. bovienii* YL002, and then their optimal concentrations were obtained by using response surface methodology. It was shown that the RSM model was adequate to predict the optimization of antibiotic production. Under optimal condition, the predicted value of the antibiotic activity was increased to 305.55 U/mL. Validation experiments were also carried out to verify the availability and the accuracy of the models, and the results showed that the predict values agreed with the experimental values well. The optimization of the medium resulted not only in a 37.8% higher antibiotic activity than media previously cited in the literature but also in a reduction of constituents costs. The chosen method of optimization of conditions was efficient, simple and less time consuming. Furthermore, the information obtained is considered fundamental and useful for the development of *X. bovienii* YL002 cultivation process for efficient production of antibiotic on a large scale.

## Materials and methods

### Symbiotic bacterium strain and culture conditions

*Xenorhabdus bovienii* YL002 was isolated from its nematode symbiont *Steinernema* sp. YL002 obtained from the soil from Yangling, China, and had been identified to be *X. bovienii* according to its morphological and molecular characteristics [36,37]. The entomopathogenic nematode-symbiotic bacteria including *X. bovienii* have two phenotypic (previous name: phase) variants, called primary form (1°) and secondary form (2°). Only 1° exhibits a considerable antibiotic activity [38]. *X. bovienii* YL002 was maintained on nutrient agar (NA) slants and subcultured monthly. The primary-secondary switch is spontaneous and in case of *X. bovienii* is irreversible. Consequently the laboratory cultures should be scored after recovering from the 20% glycerol suspension in which they had been stored in the deep freezer. To ensure the predominance of 1° population, refrozen cells were seeded on the surface of NA media supplemented with 0.04% triphenyltetrazolium chloride (w/v) and 0.025% bromothymol blue (w/v) (NBTA), and fresh liquid culture were started from single blue (1°) colonies as described [39].

Seed culture for antibiotic production was prepared by inoculating a loopful of phase I *X. bovienii* YL002 from a 72 h culture growing on an NBTA plate into a 250 mL flask containing 50 mL fresh YSG medium (glycerol 5 g/L, yeast extract 15 g/L, 1 M MgSO_4_ 5 mL, (NH_4_)_2_SO_4_ 2 g/L, 1 M KH_2_OP_4_ 5 mL, 1 M K_2_HOP_4_ 5 mL and 1 M Na_2_SO_4_ 10 mL) [19], which was used in all seeding experiments. Media were adjusted to a final pH of 7.0 with 1 M NaOH solution to provide optimal condition of growth for *X. bovienii* YL002. The flasks were incubated in the dark at 28°C on an Eberbach rotary shaker at 150 rpm for 18-24 h until the optical density (600 nm) and pH readings were approximately 2.0 and 7.0, respectively.

### Assay of antibiotic activity

Antibiotic activity was measured by agar diffusion plate assay with *Bacillus subtilis*[40]. Briefly, 1 mL containing 10^7^-10^8^ colonies of *B. subtilis* was applied to NA plate. After 2 h incubation at room temperature, the supernatants of culture following microfilteration (100 μL), using a 0.22 μm syringe microfilter, were placed on 6-mm disk filters (Whatman 3-mm paper) and air dried. The dried disks were put on the NA plate and incubated at 28°C for 24 h to determine the relationship between the size of the zones of inhibited bacterial growth and the concentration of the antibiotic. Zones of inhibition were measured, and measurements were taken from the edge of antibiotic disk to the margin of the zone of inhibition. Antibiotic activity was expressed as units of activity per milliliter the supernatants of culture, where 1 U was defined as a 1.0 mm annular clearing around the antibiotic disk.

Maxwell et al. confirmed the assumption that the changes in the size of the zones of inhibition (expressed as units of activity per gram of insect tissue) represented changes in antibiotic concentration; the antibiotics were extracted from insect larvae killed by *X. bovienii* by homogenizing the insects in distilled water [40]. The assumption has been used successfully to measure the antibiotic activity of *X. nematophila* YL001 [41]. Therefore, the size of the zones of inhibition served as a measure of antibiotic titer of *X. bovienii* YL002.

### Measurement of growth

Cell growth was measured by optical density of the culture at 600 nm and biomass concentrations (DCW: g/L) were determined using a calibration curve. The calibration curve was calculated using dilutions of a biomass suspension with known optical density. A fixed volume of the dilutions was centrifuged (20 mins, 4°C at 22400 g, Himac CR 22G, Japan) and drying of the cell pellets at 110°C for 24 h. All the cell pellets were weighed before the centrifugation and after the drying. Thus, a relationship between biomass concentration (g/L) and optical density (600 nm) can be determined.

### Selection of the optimal nutrient medium for antibiotic production

Different media such as nutrient broth, modified nutrient broth (beef extract 3 g/L, peptone 5 g/L, NaCl 10 g/L), PP3 broth (proteose peptone 20 g/L), modified PP3 broth (proteose peptone 20 g/L, NaCl 10 g/L), LB (tryptone 10 g/L, yeast extract 5 g/L, NaCl 10 g/L), YS broth (yeast extract 5 g/L, (NH_4_)_2_SO_4_ 5 g/L, MgSO_4_·7H_2_O 0.2 g/L, KH_2_PO_4_ 0.5 g/L, K_2_HPO_4_ 0.5 g/L), TSB (tryptone 17 g/L, soytone 3.0 g/L, glucose 2.5 g/L, NaCl 5.0 g/L) and YSG were used in comparative studies to find the optimal nutrient medium for antibiotic production. Five microlitter of the seed culture was transferred into 50 mL of different sterile medium in 500 mL flask. The flasks were incubated in the dark at 25°C on an rotary shaker at 150 rpm. Samples of ca. 3 mL were withdrawn each 6 h approximately. An amount of 1 mL aliquots of the fermentation broth were centrifuged (20 mins, 4°C at 26880 g, Himac CR 22G) to separate the bacteria cells from the supernatants. The supernatants were store at 4°C until required for use.

### Selection of best carbon and nitrogen sources

In order to investigate the effect of the carbon and nitrogen sources on the antibiotic activity of *X. bovienii* YL002, the optimal nutrient medium for antibiotic production was employed as an original medium for the following optimization studies. Various simple and complex carbon and nitrogen sources (Figure 2) were used individually instead of the corresponding carbon and nitrogen sources in the optimal nutrient medium while other components were kept constant at original concentration, and the antibiotic activity was determined after 72 h of incubation at 25°C under shaking (150 rpm).

### Experimental design and optimization by RSM

The response surface methods by using a set of experimental design (Central composite experimental design) were used to optimize the constituents of antibiotic production. The selected independent variables of the medium components were glycerol (*X*_1_), soytone (*X*_2_) and minerals (*X*_3_) defined as the total concentration of the minerals of YSG medium and kept the proportion of diversified components. According to the central composite design, the total number of experimental combinations is 2*^k^* + 2*k* + *n*_0_, where *k* is the number of independent variables and *n*_0_ is the number of repetitions of the experiments at the centre point [42]. For statistical calculation, the experimental variables *X*_i_ have been coded as *x*_i_ according to the following transformation equation:

(1) $$x_{i}=\frac{X_{i}-{\bar{X}}_{i}}{\Delta X_{i}}\;\left(i=1,\;2,\;3,\;\cdot \cdot \;\cdot \cdot k\right)$$

where *x*_i_ is the independent variable coded value, *X*_i_ is the independent variable real value, ${\bar{X}}_{i}$is the independent variable real value on the centre point, and Δ*X*_i_ is the step change value. The response variable (antibiotic activity units) was fitted by a second order model in order to correlate the response variable to the independent variables. The general form of the second degree polynomial equation is

(2) $$Y=\beta_{0}+\underset{i}{\sum }\beta_{i}x_{i}+\underset{i}{\sum }\underset{j}{\sum }\beta_{ij}x_{ij}+\sum \beta_{ii}{x_{i}}^{2}$$

where *Y* is the measured response, *β_0_ is* the intercept term, *β_i_* , *β_ij_*, and *β_ii_* are the measures of the effects of variables *x_i_*, *x_i_x_j_* , and *x*^2^*_i_*, respectively. The variable *x_i_x_j_* represents the first-order interaction between *x_i_* and *x_j_* (*i* <*j*).

For the medium constituent optimization, a 2^3^-factorial experimental design (CCD) for three independent variables each at five levels with four axial points and six replicates at the center points leading to a total number of twenty experiments was employed for the optimization of the medium constituents. The STATISTICA 8.0 software (StatSoft Inc. Tulsa, the USA) was used for regression and graphical analyses of the data obtained. The response variable was assigned at low and high of the observed values for a desirability of 0 and 1, respectively, to get the overall desirability. The desirability function to get the optimum combinations of independent variables was fitted by the least square method using the software. The 3D response graph and profile for predicted values and desirability level for factors were plotted using the same software.

The statistical analysis of the model was performed in the form of analysis of variance (ANOVA). This analysis included the Fisher's *F*-test (overall model significance), its associated probability *P*(*F*), correlation coefficient *R*, and determination coefficient *R*2 that measures the goodness of fit of regression model. The analysis also included the Student's *t*-value for the estimated coefficients and associated probabilities, *P*(*t*). For each variable, the quadratic models were represented as contour plots.

## Competing interests

The authors declares that they have no competing interest.

## Authors' contributions

YW conceived, designed and organized the study. XF conducted the data analysis. YW and XF conducted the interpretation of results and drafted the manuscript. YW and XF contributed equally to this work as first authors. FA and GW carried out the experiments. XZ participated in critical reviews and improvement of the manuscript. All authors have read and approved the final manuscript.

## Acknowledgements

This work was supported by the Natural Science Foundation of China (No. 31171910), National Department Public Benefit Research Foundation of China (No. 200903052) and Young People Science Program of Northwest A & F University (No. 52211241).

## References

[B1] Forst S Nealson KH Molecular biology of the symbiotic-pathogenic bacteria *Xenorhabdus* spp. and *Photorhabdus* spp Microbiol Rev 1996 60 21 43 8852894 10.1128/mr.60.1.21-43.1996 PMC239416

[B2] Akhurst RJ Antibiotic activity of *Xenorhabdus* spp., bacteria symbiotically associated with insect pathogenic nematodes of the families *Hettrorhabditidae* and *Steinernematidae* J Gen Microbiol 1982 128 3061 3065 7183749 10.1099/00221287-128-12-3061

[B3] Chen G Dunphy GB Webster JM Antifungal activity of two *Xenorhabdus* species and *Photorhabdus luminescens*, bacteria associated with the nematodes *Steinernema* species and *Heterorhabditis megidis* Biol Control 1994 4 157 162 10.1006/bcon.1994.1025

[B4] Chen G Maxwell P Dunphy GB Webster JM Culture conditions for *Xenorhabdus* and *Photorhabdus* symbionts of entomopathogenic nematodes Nematologica 1996 42 124 127 10.1163/187529296X00139

[B5] Gaugler R Kaya HK Entomopathogenic nematodes in biological control 1990 Boca Raton Florida, USA, CRC Press 75 90

[B6] Paul VJ Frautschy S Fenical W Nealson KH Antibiotics in microbial ecology, isolation and structure assignment of several new antibacterial compounds from the insect symbiotic bacteria *Xenorhabdu*s spp J Chem Ecol 1981 7 589 597 10.1007/BF00987707 24420598

[B7] McInerney BV Gregson RP Lacey MJ Akhurst RJ Lyons GR Rhodes SH Smith DRJ Engelhardt LM Biologically active metabolites from *Xenorhabdus* spp. Part 1. Dithiolopyrrolone derivatives with antibiotic activity J Nat Prod 1991 54 774 784 10.1021/np50075a005 1955880

[B8] McInerney BV Taylor WC Lacey MJ Akhurst RJ Gregson RP Biologically active metabolites from *Xenorhabdus* spp Part 2 Benzopyran-1-one derivatives with gastroprotective activity J Nat Prod 1991 54 785 795 10.1021/np50075a006 1955881

[B9] Li JX Chen GH Webster JM Nematophin, a novel antimicrobial substance produced by *Xenorhabdus nematophilus* (Enterobactereaceae) Can J Microbiol 1997 43 770 773 10.1139/m97-110 9304787

[B10] Ji DJ Yi YK Kang GH Identification of an antibacterial compound, benzylideneacetone, from *Xenorhabdus nematophila* against major plant-pathogenic bacteria FEMS Microbiol Lett 2004 239 241 248 10.1016/j.femsle.2004.08.041 15476972

[B11] Lang G Kalvelage T Peters A Wiese J Imhoff JF Peptides from the entomopathogenic bacterium *Xenorhabdus nematophilus* J Nat Prod 2008 71 1074 1077 10.1021/np800053n 18491867

[B12] Gualtieri M Aumelas A Thaler JO Identification of a new antimicrobial lysine-rich cyclolipopeptide family from *Xenorhabdus nematophila* J Antibiot 2009 62 295 302 10.1038/ja.2009.31 19373275

[B13] Li JX Chen GH Webster JM Czyzewska E Antimicrobial metabolites from a bacterial symbiont J Nat Prod 1995 58 1081 1086 10.1021/np50121a016 7561900

[B14] Chen G Antimicrobial activity of the nematode symbionts, *Xenorhabdus* and *Photorhabdus* (Enterobacteriaceae) and the discovery of two novel groups of antimicrobial substances, nematophin and xenorxides PhD thesis 1996 Simon Fraser University, British Columbia, Canada

[B15] Ng KK Webster JM Antimycotic activity of *Xenorhabdus bovienii* (Enterobacteriaceae) metabolites against *Phytophthora infestans* on potato plants Can J Plant Pathol 1997 19 125 132 10.1080/07060669709500540

[B16] Shapiro-Ilan DI Reilly CC Hotchkiss M Suppressive effects of metabolites from *Photorhabdus* and *Xenorhabdus* spp. on phytopathogens of peach and pecan Arch Phytopathol Plant Prot 2009 42 715 728 10.1080/03235400701390539

[B17] Fang XL Li ZZ Wang YH Zhang X *In vitro* and *in vivo* antimicrobial activity of *Xenorhabdus bovienii* YL002 against *Phytophthora capsici* and *Botrytis cinerea* J Appl Microbiol 2011 111 145 154 10.1111/j.1365-2672.2011.05033.x 21554568

[B18] Scherlach K Hertweck C Triggering cryptic natural product biosynthesis in microorganisms Org Biomol Chem 2009 7 1753 1760 10.1039/b821578b 19590766

[B19] Sundar L Chang FN Antimicrobial activity and biosynthesis of indole antibiotics produced by *Xenorhabdus nematophilus* J Gen Microbiol 1993 139 3139 3148 7510325 10.1099/00221287-139-12-3139

[B20] Doull JL Vining LC Nutritional control of actinorhodin production by *Streptomyces coelicolor* A3(2): suppressive effects of nitrogen and phosphate Appl Microbiol Biotechnol 1990 32 449 454 10.1007/BF00903781 1366394

[B21] Duchaud E Rusniok C Frangeul L Buchrieser C Givaudan A Taourit S Bocs S Boursaux-Eude C Chandler M Charles JF The genome sequence of the entomopathogenic bacterium *Photorhabdus luminescens* Nat Biotechnol 2003 21 1307 1313 10.1038/nbt886 14528314

[B22] Bode HB Bethe B Hofs R Zeeck A Big effects from small changes: possible ways to explore nature's chemical diversity Chem Bio Chem 2002 3 619 627 12324995 10.1002/1439-7633(20020703)3:7<619::AID-CBIC619>3.0.CO;2-9

[B23] Rosso1 AM Ferrarotti1 SA Krymkiewicz1 N Nudel BC Optimisation of batch culture conditions for cyclodextrin glucanotransferase production from *Bacillus circulans* DF 9R Microb Cell Fact 2002 1 3 10.1186/1475-2859-1-3 12392599 PMC140141

[B24] Barghini1 P Gioia DD Fava F Ruzzi M Vanillin production using metabolically engineered *Escherichia coli* under non-growing conditions Microb Cell Fact 2007 6 13 10.1186/1475-2859-6-13 17437627 PMC1857700

[B25] Cheng HR Wang HW Lv JY Jiang MG Lin SJ Deng ZX A novel method to prepare L-Arabinose from xylose mother liquor by yeast-mediated biopurification Microb Cell Fact 2011 10 43 10.1186/1475-2859-10-43 21649890 PMC3125199

[B26] Ongkudon CM Pickering R Webster D Danquah MK Cultivation of *E. coli* carrying a plasmid-based Measles vaccine construct (4.2 kbp pcDNA3F) employing medium optimisation and pH-temperature induction techniques Microb Cell Fact 2011 10 16 10.1186/1475-2859-10-16 21375765 PMC3059270

[B27] Wang YH Li YP Zhang Q Zhang X Enhanced antibiotic activity of *Xenorhabdus nematophila* by medium optimization Bioresour Technol 2008 99 1708 1715 10.1016/j.biortech.2007.03.053 17531470

[B28] Sleator RD Hill C Bacterial osmoadaptation: The role of osmolytes in bacterial stress and virulence FEMS Microbiol Rev 2002 26 49 71 10.1111/j.1574-6976.2002.tb00598.x 12007642

[B29] Crawford JM Kontnik R Clardy J Regulating alternative lifestyles in entomopathogenic bacteria Curr Biol 2010 20 69 74 10.1016/j.cub.2009.10.059 20022247 PMC2821981

[B30] Krasomil-Osterfeld KC Influence of osmolarity on phase shift in *Photorhabdus luminescens* Appl Environ Microbiol 1995 61 3748 3749 16535154 10.1128/aem.61.10.3748-3749.1995 PMC1388716

[B31] Yang XF Qiu DW Jiao NN Liu Z Yuan JJ Cultural medium and fermentation conditions of *Xenorhabdus* sp. Strain D43 Chin J Biol Control 2006 22 58 62

[B32] Yang XF Yang HW Jian H Liu Z Effect of fermentation conditions on antibiotic production of *Xenorhabdus nematophilus* Chin Microbiol 2001 28 12 16

[B33] Fang XL Feng JT Zhang WG Wang YH Zhang X Optimization of growth medium and fermentation conditions for improved antibiotic activity of *Xenorhabdus nematophila* TB using a statistical approach Afr J Biotechnol 2010 9 8068 8077

[B34] Roberto P David A Naresh M Medium optimization for the production of the secondary metabolite squalestatin S1 by a *Phoma* sp. combining orthogonal design and response surface methodology Enzyme Microb Technol 2005 37 704 711 10.1016/j.enzmictec.2005.04.009

[B35] Thella JS Venugopal R Modeling of iron ore pelletization using 3 **^(k-p)^ factorial design of experiments and polynomial surface regression methodology Powder Technol 2011 211 54 59 10.1016/j.powtec.2011.03.027

[B36] Wang YH Zhang X Isolation and identification of symbiotic bacteria associated with entomopathogenic nematodes J Northwest A & F Univ 2006 34 174 18022044452

[B37] Fang XL Guo Q Yi XH Wang YH Zhang X Molecule identification and cultivation characteristic of the bacterial symbiont of the entomopathogenic nematode J Northwest A & F Univ 2008 36 200 20422044452

[B38] Boemare NE Akhurst RJ Biochemical and physiological characterization of colony form variants in *Xenorhabdus* spp. (Enterobacteriaceae) J Gen Microbiol 1988 134 751 761 10.1099/00221287-134-7-1835 3246587

[B39] Lengyel K Lang E Fodor A Szállás E Schumann P Stackebrandt E Description of four novel species of *Xenorhabdus*, family Enterobacteriaceae: Xenorhabdus budapestensis sp. nov., Xenorhabdus ehlersii sp. nov., Xenorhabdus innexi sp. nov., and Xenorhabdus szentirmaii sp. nov Syst Appl Microbiol 2005 28 115 122 10.1016/j.syapm.2004.10.004 15830803

[B40] Maxwell PW Chen G Webster JM Dunphy GB Stability and activities of antibiotics produced during infection of the insect *Galleria mellonella* by two isolates of *Xenorhabdus nematophilus* Appl Environ Microbiol 1994 60 715 721 16349198 10.1128/aem.60.2.715-721.1994 PMC201371

[B41] Wang YH Fang XL Li YP Zhang X Effects of constant and shifting dissolved oxygen concentration on the growth and antibiotic activity of *Xenorhabdus nematophila* Bioresour Technol 2010 101 7529 7536 10.1016/j.biortech.2010.04.070 20488698

[B42] Box GEP Wilson KB On the experimental attainment of optimum conditions J Roy Stat 1951 13 1 45

